# Das AKTIN-Notaufnahmeregister – kontinuierlich aktuelle Daten aus der Akutmedizin: Ergebnisse des Registeraufbaus und erste Datenauswertungen aus 15 Notaufnahmen unter besonderer Berücksichtigung der Vorgaben des Gemeinsamen Bundesausschusses zur Ersteinschätzung

**DOI:** 10.1007/s00063-020-00764-2

**Published:** 2020-12-21

**Authors:** D. Brammen, F. Greiner, M. Kulla, R. Otto, W. Schirrmeister, S. Thun, S. E. Drösler, J. Pollmanns, S. C. Semler, R. Lefering, V. S. Thiemann, R. W. Majeed, K. U. Heitmann, R. Röhrig, F. Walcher, AKTIN-Notaufnahmeregister, Jonas Bienzeisler, Corinna Feeken, Hauke Fischer, Benjamin Lucas, Tolga Nazyok, Tingyan Xu, Jörg Brokmann, Carsten Mach, Caroline Grupp, Stefan Kühner, Christian Pietsch, Oliver Horn, Christian Wrede, Heike Höger-Schmidt, Harald Dormann, Sabine Blaschke, Sönke Bax, Wilhelm Behringer, Markus Rettig, Rupert Grashey, Thomas Henke, Kirsten Habbinga, Tobias Schilling, Eckart Wetzel, Markus Baacke, Bernadett Erdmann

**Affiliations:** 1 Universitätsklinik für Unfallchirurgie, Otto-von-Guericke-Universität Magdeburg, Leipziger Str. 44, 39120 Magdeburg, Deutschland; 2 Universitätsklinik für Anästhesiologie und Intensivtherapie, Otto-von-Guericke-Universität Magdeburg, Magdeburg, Deutschland; 3 Klinik für Anästhesiologie, Intensivmedizin, Notfallmedizin und Schmerztherapie, Bundeswehrkrankenhaus Ulm, Ulm, Deutschland; 4 Competence Center eHealth, Hochschule Niederrhein, Krefeld, Deutschland; 5 Fachbereich Gesundheitswesen, Hochschule Niederrhein, Krefeld, Deutschland; 6 TMF – Technologie- und Methodenplattform für die vernetzte medizinische Forschung e. V., Berlin, Deutschland; 7 Institut für Forschung in der Operativen Medizin (IFOM), Universität Witten/Herdecke, Köln, Deutschland; 8 Abteilung Medizinische Informatik, Carl von Ossietzky Universität Oldenburg, Oldenburg, Deutschland; 9 Institut für Medizinische Informatik, Medizinische Fakultät, RWTH Aachen, Aachen, Deutschland; 10 Heitmann Consulting and Services, Hürth, Deutschland; 11 hih – health innovation hub, Bundesministerium für Gesundheit, Berlin, Deutschland

**Keywords:** Notaufnahme, Register, Digitalisierung, Interoperabilität, Versorgungsforschung, Emergency department, Registry, Digitalization, Health information interoperability, Health services research

## Abstract

**Hintergrund:**

Die Notfallversorgung befindet sich im Umbruch. In Notaufnahmen werden Patienten ausgehend von Symptomen und Dringlichkeit versorgt; dies bildet sich jedoch in den etablierten Routinedaten der gesetzlichen Sozialversicherung nicht ab. Ziel des AKTIN-Projekts war der Aufbau einer datenschutzkonformen Registerinfrastruktur zur Nutzung von klinischen Routinedaten aus Notaufnahmen.

**Methoden:**

Über eine standardisierte Schnittstelle werden aus den verschiedenen Dokumentationssystemen kontinuierlich Daten der Notaufnahmebehandlung in ein lokales Data Warehouse exportiert. Dort stehen sie sowohl für lokale Nutzungen, wie interne Berichte und Qualitätsmanagement, als auch gleichzeitig datenschutzkonform für multizentrische Auswertungen zur Verfügung.

Anhand der Registerpopulation wird die Ersteinschätzung sowie die Erhebung von Vitalparametern in Abhängigkeit von Vorstellungsgründen für einen 12-Monats-Zeitraum analysiert.

**Ergebnisse:**

Für den Zeitraum 04/2018 bis 03/2019 wurden 436.149 gültige Fälle aus 15 Notaufnahmen übermittelt. In 86,0 % der Fälle ist eine Ersteinschätzung dokumentiert.

Diese fand in 70,5 % innerhalb von 10 min nach Ankunft des Patienten statt. In 10 Kliniken wird regelhaft (82,3 %) ein Vorstellungsgrund erfasst. Die Erfassung von Vitalparametern variiert plausibel zwischen den Vorstellungsgründen.

**Schlussfolgerung:**

Das AKTIN-Notaufnahmeregister bietet einen zeitnahen Einblick in das Versorgungsgeschehen der Notaufnahmen ohne zusätzlichen Dokumentationsaufwand und unabhängig vom primären IT-System, Kostenträger, Fallart und Abrechnungsmodus. Die Vorgaben des Gemeinsamen Bundesausschusses zur Ersteinschätzung werden weitgehend umgesetzt. Durch die Etablierung von standardisierten Vorstellungsgründen werden symptombasierte Analysen und Gesundheitssurveillance ermöglicht.

**Zusatzmaterial online:**

Die Onlineversion dieses Beitrags (10.1007/s00063-020-00764-2) enthält die Abb. S1 und S2. Beitrag und Zusatzmaterial stehen Ihnen auf www.springermedizin.de zur Verfügung. Bitte geben Sie dort den Beitragstitel in die Suche ein, das Zusatzmaterial finden Sie beim Beitrag unter „Ergänzende Inhalte“.

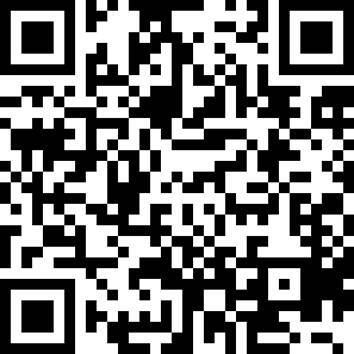

## Abstract (en)

**Background:**

Emergency care in Germany is in transition. Emergency departments (EDs) treat their patients based on symptoms and acuity. However, this perspective is not reflected in claims data. The aim of the AKTIN project was to establish an Emergency Department Data Registry as a data privacy-compliant infrastructure for the use of routine medical data.

**Methods:**

Data from the respective documentation systems are continuously transmitted to local data warehouses using a standardized interface. They are available for several applications such as internal reports but also multicentre studies, in compliance with data privacy regulations. Based on a 12-months period we evaluate the population with focus on acuity assessment (triage) and vital parameters in combination with presenting complaints.

**Results:**

For the period April 2018 to March 2019, 436,149 cases from 15 EDs were available. A triage level is documented in 86.0% of cases, and 70.5% were triaged within 10 min of arrival. Ten EDs collected a presenting complaint regularly (82.3%). The respective documentation of vital signs shows plausible patterns.

**Conclusions:**

The AKTIN registry provides an almost real-time insight into German EDs, regardless of the primary documentation system and health insurance claims data. The Federal Joint Committee’s requirements are largely met. Standardized presenting complaints allow for symptom-based analyses as well as health surveillance.

## Hintergrund

Die Versorgung von Notfallpatienten befindet sich aktuell im Umbruch. Der Beschluss des Gemeinsamen Bundesausschusses (G-BA) zu einem gestuften System von Notfallstrukturen in Krankenhäusern auf Basis des Krankenhausstrukturgesetzes sowie die geplante Reform der Notfallversorgung werden die Versorgungslandschaft in naher Zukunft nachhaltig verändern [6, 7, 33]. Abrechnungsdaten sind in den letzten Jahren eine wichtige Datengrundlage für die Versorgungsforschung geworden [8, 38]. Allerdings wird in diesen Routinedaten die Notfallversorgung nicht adäquat abgebildet, da sie nur diagnosebezogene Auswertungen zulassen und damit der Kernfunktion einer Notaufnahme, nämlich Diagnosestellung und Therapieeinleitung auf Basis von Symptomen, nicht gerecht werden [12, 14, 24]. Weiterhin wird die obligatorische Ersteinschätzung zur Priorisierung der Versorgung [6] nicht dargestellt. Gleichzeitig existiert aufgrund der sektoralen Trennung im deutschen Gesundheitswesen bisher kein Datenhalter, der eine Zusammenschau aller Notfallbehandlungen zulässt [15]. Somit wird ein Großteil der Patientenversorgung in Notaufnahmen und den zu etablierenden integrierten Notfallzentren (INZ) von den vorhandenen Datenquellen nicht erfasst. Die Behandlungsdokumentation klinisch versorgter Notfallpatienten in einem Register kann diese Lücke schließen [26].

## Zielsetzung

Ziel des vom Bundesministerium für Bildung und Forschung (BMBF) von 11/2013 bis 10/2019 geförderten Projekts „Verbesserung der Versorgungsforschung in der Akutmedizin in Deutschland durch den Aufbau eines nationalen Notaufnahmeregisters“ (AKTIN, hervorgegangen aus dem Aktionsbündnis für Informations- und Kommunikationstechnologie in Intensiv- und Notfallmedizin) war im ersten Schritt die semantische und syntaktische Standardisierung eines etablierten Dokumentationsstandards für Notaufnahmen [20, 41]. Durch die Entwicklung und Implementierung einer datenschutzkonformen Infrastruktur sollte die elektronische Behandlungsdokumentation aus Notaufnahmen ohne zusätzlichen Dokumentationsaufwand interoperabel, das heißt unabhängig vom verwendeten IT-System, einer sekundären Datennutzung zugeführt werden [1]. Primäres Ziel dieser Arbeit ist die Beschreibung der Infrastruktur des AKTIN-Notaufnahmeregisters. Sekundäres Ziel ist eine Auswertung der dokumentierten Ersteinschätzung als aktuell relevantes Beispiel für Qualitätsmanagement sowie Versorgungsforschung.

## Methodik

### Setting – teilnehmende Notaufnahmen

**Figure 1:**
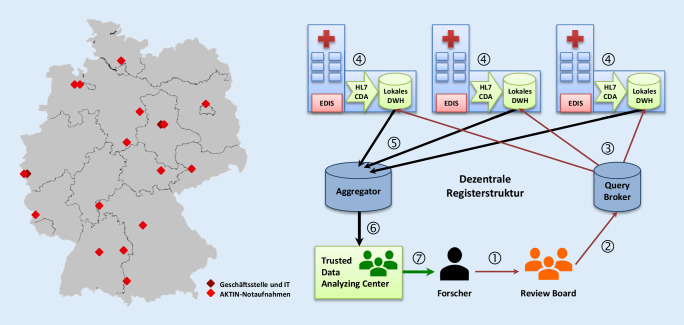

Während der geförderten Projektlaufzeit wurden 16 Notaufnahmen rekrutiert, davon eine ohne Projektförderung. Die Kliniken sind deutschlandweit verteilt (Abb. 1), versorgen jeweils zwischen 12.000 und 60.000 Fälle jährlich und decken die im G‑BA-Beschluss genannten 3 Stufen der Notfallversorgung ab [6]. Teilnahmevoraussetzung ist die elektronische Dokumentation der Patientenversorgung in einem Notaufnahmeinformationssystem (Emergency Department Information System, EDIS) und der Betrieb einer standardisierten AKTIN-Exportschnittstelle. Es existieren keine fallbezogenen Ein- oder Ausschlusskriterien seitens des Registers. Ziel ist die Vollerhebung des Patientenkollektivs in jeder Notaufnahme.

### Datensatz Notaufnahme

Der Datensatz Notaufnahme der Deutschen Interdisziplinären Vereinigung für Intensiv- und Notfallmedizin (DIVI) e. V. bezieht sich ausschließlich auf den Behandlungszeitraum in der Notaufnahme. Er definiert im Basismodul der Version 2015.1 [21] demographische Daten, einen standardisierten Vorstellungsgrund (CEDIS-Code; [4]), die Ersteinschätzung gemäß Manchester Triage System (MTS) oder Emergency Severity Index (ESI), Vitalparameter, Prozesszeiten und -parameter sowie Diagnosen (kodiert nach ICD-10-GM) zum Ende der Notaufnahmebehandlung. Für die lokale Registerdatenbank wurden zusätzlich Aufnahmedatum und -zeitpunkt sowie eine klinikinterne Identifikationsnummer für Patient und Behandlungsfall als Minimalinformationen definiert.

### Infrastruktur und Datenflüsse

Die Daten werden im Rahmen der Routinedokumentation mit dem jeweiligen EDIS dokumentiert und primär in der klinikeigenen IT-Architektur gespeichert. Der Export in ein *lokales* Data Warehouse (DWH) erfolgt über eine standardisierte, konsentierte IT-Schnittstelle (Health Level Seven Clinical Document Architecture, HL7 CDA) unter Nutzung von semantischen Interoperabilitätsstandards (ICD-10-GM, OPS, LOINC (Logical Observation Identifiers Names and Codes) sowie proprietären Ersatzcodes als Substitution für die Terminologie SNOMED CT). In den 16 AKTIN-Kliniken sind EDIS von 8 unterschiedlichen Herstellern im Einsatz (Dedalus ORBIS Notaufnahme, Bonn, Deutschland; E.care ED, Turnhout, Belgien; epias ED, Idstein, Deutschland; eHealth-Tec ERPath, Berlin, Deutschland; CGM medico, Koblenz, Deutschland; IMESO-IT ICUData, Gießen, Deutschland; COPRA, Berlin, Deutschland; Cerner i.s.h.med, Idstein, Deutschland). Die Datensätze werden zu EDIS-spezifisch definierten Zeitpunkten exportiert, beispielsweise nach einem Speichervorgang oder nach Abschluss der Notfallbehandlung. Die Aktualisierung eines Datensatzes im DWH ist möglich.

### Ethik, Datenschutz und Anonymisierung

Das Projekt wurde durch die Ethikkommission der Otto-von-Guericke-Universität Magdeburg an der medizinischen Fakultät positiv beurteilt (Votum 160/15) und ist im Deutschen Register Klinischer Studien registriert (DRKS00009805). Das Datenschutzkonzept wurde von der Arbeitsgruppe Datenschutz der TMF – Technologie- und Methodenplattform für die vernetzte medizinische Forschung e. V. positiv begutachtet und sieht eine pseudonymisierte Datenspeicherung im Organisationsbereich der Notaufnahmen für einrichtungsinterne Qualitätssicherungs- oder Forschungsmaßnahmen vor. Darüber hinaus ist eine Abfrage anonymisierter Daten über die zentrale AKTIN-Infrastruktur für krankenhausübergreifende Fragestellungen möglich, sofern diese durch ein gemischt intern und extern besetztes wissenschaftliches Gremium (Review Board) auf Wissenschaftlichkeit, Durchführbarkeit, Ethik und Datenschutz geprüft wurde [1]. Datenanfragen sind organisatorisch und technisch von der Datensammlung getrennt, die Aggregation und Auswertung der abgefragten Daten erfolgt in einem „trusted data analyzing center“. Anfragen von extern sind möglich, in diesem Fall werden nur aggregierte bzw. hinreichend vergröberte Daten weitergegeben (Abb. 1).

Zusätzlich entscheidet in jeder Notaufnahme ein Verantwortlicher über die Teilnahme an der jeweiligen Datenlieferung. Bei wiederkehrenden Abfragen (z. B. monatliche Erstellung von Benchmark-Berichten) ist eine einmalige, widerrufbare Zustimmung möglich.

Individuelle Patienteneinwilligungserklärungen sind im Kontext der Notfallsituation und bei den technisch-organisatorischen Maßnahmen gemäß Datenschutzkonzept nicht vorgesehen, jedoch existiert für die Patienten eine Opt-Out-Möglichkeit mit Datenlöschung.

### Datenbasis der Auswertung

In die Auswertung (Zeitraum 04/2018 bis 03/2019) fließen fallbasiert Alter in Jahren, Geschlecht, Vorstellungsgrund (CEDIS-Code), Stufe und System der Ersteinschätzung (Triage), Zeitpunkt von Aufnahme und Ersteinschätzung sowie ausgewählte Vitalwerte ein.

Nach Bereinigung um Dubletten erfolgt eine Deskription von Fallzahlen sowie ein Vergleich der Alters- und Geschlechtsverteilung mit der Bevölkerungsstruktur in Deutschland zum Stand 31.12.2018 (Quelle: Statistisches Bundesamt, Destatis). Weiterhin werden die Durchführung und das Ergebnis der Ersteinschätzung sowie das verwendete Ersteinschätzungssystem berichtet. Die Dauer zwischen Aufnahme und Ersteinschätzung wird wie folgt kategorisiert: Ersteinschätzung ist erster dokumentierter Zeitpunkt (t0) im System, >0 bis 10, >10 bis 20, >20 bis 180 und >180 min. Werte >180 min werden als nicht plausibel aus der Mittelwertberechnung ausgeschlossen. Ein Vergleich zwischen den Kliniken erfolgt zur Wahrung deren Anonymität nur anhand relativer Häufigkeiten.

Die Erhebungsquote von Vitalwerten wird in Abhängigkeit von ausgewählten Vorstellungsgründen, z. B. Luftnot (CEDIS-Code 651), Brust- (003, 004), Bauch- (251) und Rückenschmerzen (551), ausgewertet.

Um echte Fallzahlschwankungen in den einzelnen Notaufnahmen von Unterbrechungen in der Datenübermittlung differenzieren zu können, wurde eine monatliche Abweichung von ±20 % des klinikspezifischen Jahresdurchschnitts definiert.

## Ergebnisse

### Strukturaufbau

Die lokale AKTIN-Software ist seit 04/2016 betriebsfähig, die Datenübermittlung an die AKTIN-DWH innerhalb der Notaufnahmen begann 05/2016. Ein krankenhausinternes Reporting wurde 03/2017 in Betrieb genommen. Dabei wird monatsweise ein standardisierter Bericht erstellt, der Fallzahlen, Prozesszeiten, Vorstellungsgründe und Diagnosen ausweist [39].

Die zentrale, verschlüsselt kommunizierende Infrastruktur für verteilte Abfragen aus den Kliniken ist seit 04/2017 betriebsfähig [22, 39]. Die ersten einrichtungsübergreifenden Datenabfragen wurden 11/2017 durchgeführt. Ein einrichtungsübergreifendes Benchmarking besteht seit 05/2018 und wird auf monatlicher Basis an die Notaufnahmen übermittelt [28]. Ein automatisches, zentrales Monitoring überwacht seit 07/2019 den Datenimport in die lokalen AKTIN-DWH und alarmiert bei Abbruch des Datenimports oder bei Überschreitung einer definierten Fehlerquote.

### Ergebnisse der Daten aus 12 Monaten

**Figure 2:**
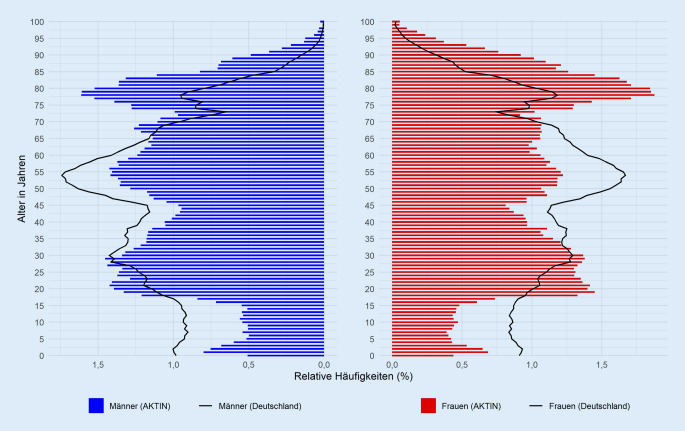

Die Datenabfrage und -auswertung wurde 03/2019 beantragt (Projekt-ID 2019-002) und nach Zustimmung durch das wissenschaftliche Gremium umgesetzt. Die Datenlieferung war innerhalb von 3 Monaten nach initialer Antragstellung abgeschlossen; eine der Kliniken hat bei unvollständiger elektronischer Datenerhebung nicht teilgenommen. Für den ausgewählten Zeitraum (04/2018 bis 03/2019) wurden 437.973 Fälle aus den 15 Notaufnahmen gesendet. Nach Löschung von 1824 Dubletten (0,4 %) wurden 436.149 Fälle ausgewertet. Im Mittel wurden monatlich 36.346 Fälle übermittelt. Aus 10 Notaufnahmen kamen monatlich konstante Fallzahlen, in 5 Notaufnahmen wurde die definierte Schwankungsbreite im Jahresverlauf überschritten (Abb. S1 und S2 im elektronischen Zusatzmaterial online). Männer (51,8 %) waren mit 48,5 Jahren im Mittel (Median: 50) etwas jünger als Frauen (48,1 %) mit 51,6 Jahren (Median 53). Bei 615 Fällen (0,1 %) war die Geschlechtsangabe nicht eindeutig. Im Vergleich mit der Bevölkerungsstruktur ist die Patientenpopulation eher älter und es wurden deutlich weniger Kinder in den AKTIN-Notaufnahmen versorgt (Abb. 2).

#### Ersteinschätzung

**Figure 3:**
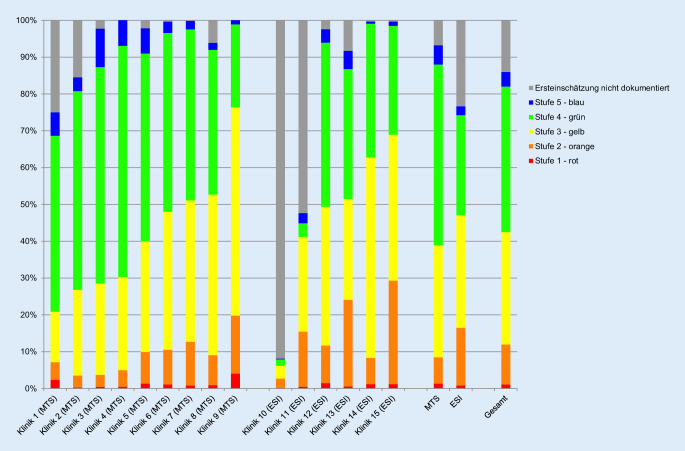

In allen Kliniken wird eine Ersteinschätzung durchgeführt, in 9 Notaufnahmen mit MTS, 6 Notaufnahmen nutzen ESI. Für 374.989 Fälle (86,0 %) liegen ein Zeitpunkt und das Ersteinschätzungsergebnis vor. Sowohl Anteil der Fälle mit Ersteinschätzung als auch Verteilung der Kategorien schwanken zwischen den Kliniken stark (Abb. 3).

Zwei Kliniken wurden von der Prozesszeitenauswertung ausgeschlossen, da offensichtlich falsche Zeitstempel übermittelt wurden. Für 340.671 der verbleibenden 401.229 Fälle liegt eine Prozesszeit vor. Bei 8,0 % dieser Fälle war der dokumentierte Zeitpunkt der Ersteinschätzung als t0 der erste Zeitpunkt im System und lag damit vor der administrativen Aufnahme, in weiteren 62,5 % fand die Ersteinschätzung innerhalb von 10 min nach administrativer Aufnahme statt. Tab. 1 zeigt die Auswertung der Zeitspannen zwischen Aufnahme und Ersteinschätzung.

**Table 1:** 

Klinik	Mittelwert^a^ (Minuten)	Median^a^ (Minuten)	Triage = t0 (in %)	>0 bis 10 min (in %)	>10 bis 20 min (in %)	>20 bis 180 min (in %)	>180 min^a^ (in %)	Gesamt (in %)
A	1,65	1,00	34,9	63,9	0,8	0,3	0,0	100,0
B	4,02	1,95	0,2	92,6	4,7	2,5	0,1	100,0
C	4,31	1,23	5,6	83,6	5,9	4,8	0,1	100,0
D	5,27	2,00	7,2	77,3	11,4	4,0	0,0	100,0
E	6,95	5,12	14,9	63,8	15,9	5,4	0,0	100,0
F	9,04	6,92	0,0	68,2	23,9	7,9	0,0	100,0
G	11,09	8,00	0,2	61,7	26,4	11,6	0,1	100,0
H	11,30	4,00	11,8	62,7	11,7	10,4	3,4	100,0
I	12,68	9,27	5,3	48,1	28,1	18,5	0,1	100,0
J	12,70	9,00	0,1	56,8	26,0	17,1	0,0	100,0
K	16,14	11,63	0,0	42,5	32,8	24,6	0,0	100,0
L	27,62	18,00	1,2	31,3	22,6	43,5	1,3	100,0
M	34,52	21,00	0,3	26,9	18,7	46,2	8,0	100,0
**Gesamt**	**10,22**	**5,00**	**8,0**	**62,5**	**16,4**	**12,3**	**0,9**	**100,0**

Triage = t0: Ersteinschätzung ist erster dokumentierter Zeitpunkt (t0) im System

^a^Werte >180 min wurden als nicht plausibel aus Berechnung von Mittelwert und Median ausgeschlossen

#### Vorstellungsgründe und Vitalparameter

**Figure 4:**
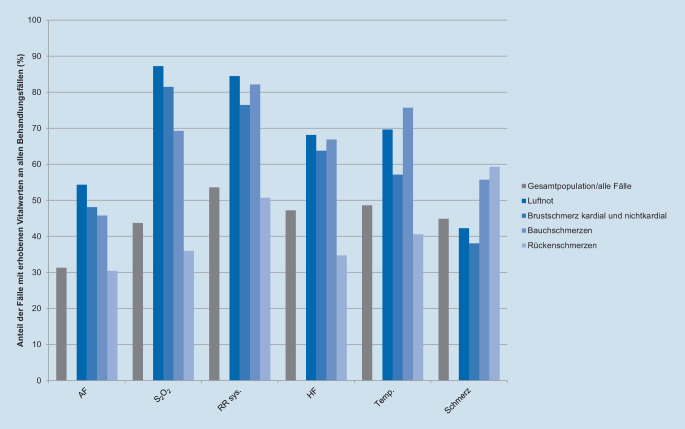

In 10 von 15 Kliniken wird regelhaft ein CEDIS-Vorstellungsgrund erfasst; die Dokumentationsquote liegt in diesen Kliniken bei 82,3 % (*n* = 255.235) von 310.285 Fällen. Als Behandlungsanlässe wurden vorrangig Verletzungen und Schmerzen der Extremitäten (*n* = 59.756, 19,3 %) Bauchschmerzen (*n* = 21.189, 6,8 %), Brustschmerz (kardial und nichtkardial; *n* = 12.295, 4,0 %), Luftnot (*n* = 9623, 3,1 %) sowie Riss‑/Quetsch‑/Schnitt‑/Stichwunden (*n* = 9473, 3,1 %) erhoben. Abb. 4 zeigt die Dokumentationshäufigkeit von relevanten Vitalparametern und Schmerzintensität (numerische Rating-Skala mit Werten von 0–10) für die Vorstellungsgründe Luftnot, Brustschmerz, Bauchschmerzen und Rückenschmerzen (*n* = 8947, 2,9 %).

## Diskussion

### Aufbau der Registerstruktur

Die Ergebnisse zeigen anhand von 15 Notaufnahmen aller Versorgungsstufen und 8 IT-Systemen, dass das entwickelte AKTIN-Notaufnahmeregister grundsätzlich geeignet ist, um die klinische Routinedokumentation zeitnah während oder spätestens bei Abschluss der Dokumentation verfügbar zu machen. Durch die Nutzung eines standardisierten Datensatzes und einer interoperablen Schnittstelle können Daten aus verschiedenen Dokumentationssystemen zusammengeführt und standardisiert ausgewertet werden. Im Gegensatz zu anderen Registern entfällt die redundante Datenerfassung bzw. manuelle Übertragung von Daten in registerspezifische Dokumentationsbögen [34]. Die Weiterleitung von Daten aus elektronischen Dokumentationssystemen an Register wird seit längerer Zeit intensiv beforscht [5, 25, 29]. Das Grundprinzip der dezentralen Datenhaltung in lokalen Data-Warehouse-Systemen wird auch in den Konsortien der BMBF-Förderinitiative Medizinische Informatik verfolgt [30]. Das konzeptionell vergleichbare pädiatrische Notaufnahmeregister (PECARN, USA) nutzt ebenfalls Daten aus EDIS, hat aber nur 7 Notaufnahmen und Systeme von 2 IT-Herstellern angebunden [11]. Im Gegensatz zu PECARN mit einer proprietären, projektspezifischen XML-Schnittstelle nutzt das AKTIN-Notaufnahmeregister mit einem HL7 CDA einen vielfach geforderten syntaktischen und semantischen Interoperabilitätsstandard [27]. Damit stehen die Daten der Notaufnahmeversorgung für zusätzliche Anwendungen in unterschiedlichen IT-Systemen zur Verfügung, auch wenn die primär geplante Nutzung der Terminologie SNOMED CT wegen lizenzrechtlicher Bedenken in Deutschland trotz Vorbereitung und Kodierung aller Begriffe im Rahmen der geförderten Projektlaufzeit nicht weiterverfolgt werden konnte [3].

International basieren die meisten Benchmarking-Initiativen in der Notfallmedizin auf administrativen Routinedaten [9, 13, 31]. Allerdings können derartige Abrechnungsdaten die klinisch relevante und patientenzentrierte Sicht auf die Versorgung nur bedingt abbilden [10]. Benchmarking anhand klinischer Daten hingegen basieren oft auf manueller Datenerhebung [36, 37], die hier gefundene Lösung hat damit Vorbildcharakter.

### Charakteristika der versorgten Patienten

Die Fallzahlen der 15 teilnehmenden Notaufnahmen befinden sich im Rahmen publizierter Daten [35], die Altersverteilung der Patienten wird oft nur monozentrisch erhoben [14, 32]. Ein Bezug zur Basispopulation wird eher selten hergestellt, eine Ausnahme wurde von Trentzsch et al. für das Notaufnahmeaufkommen in der Stadt München publiziert [40]. Kinder und Jugendliche werden häufiger in eigenen Notaufnahmen der pädiatrischen Kliniken versorgt [40]. Unter der Annahme, dass alle Patienten einer Notaufnahme im EDIS dokumentiert werden, kann von einer Vollerhebung unabhängig von Fallart, Abrechnungsmodus und Kostenträger ausgegangen werden. Eine Beeinflussung durch administrative Vorgaben (z. B. Fallzusammenführungen) ist so nicht gegeben [15]. Diese klinische Perspektive ist mit den Datensätzen der Kostenträger (z. B. Daten gemäß §§ 295 bzw. 301 Sozialgesetzbuch V) nicht darstellbar.

### Dokumentation der Ersteinschätzung

Eine durchgeführte Ersteinschätzung wurde in 86 % aller Fälle dokumentiert. Während des Untersuchungszeitraums wurde die Ersteinschätzung in 2 Notaufnahmen erst eingeführt. Die Prozesszeiten lassen sich nur bei Kenntnis der lokalen Gegebenheiten bewerten, da der Ablauf zwischen Ankunft, administrativer Aufnahme und Ersteinschätzung in den Häusern unterschiedlich geregelt ist. In einigen Notaufnahmen werden kritisch kranke Patienten unmittelbar ohne Ersteinschätzung versorgt. Eine retrospektive „Ersteinschätzung“ nach Behandlungsbeginn ist obsolet und würde einen unnötigen Ressourcenaufwand [23] darstellen. Da der G‑BA eine Ersteinschätzung *aller* Patienten fordert, wird diese dennoch teilweise bis zu Stunden später nachgeholt bzw. dokumentiert. Diese nachträgliche Ersteinschätzung erzeugt zwar eine vollständige Ersteinschätzungsquote, führt aber bei fehlender „Rückdatierung“ des Zeitstempels zu nicht validen Prozesszeiten. Dies erklärt die teilweise exzessiven Überschreitungen der vom G‑BA vorgegebenen 10 min. Eine gewisse Quote nichterfolgter Ersteinschätzungen sollte eher als Qualitätsmerkmal für die Patientenorientierung einer Notaufnahme angesehen werden.

### Dokumentation von Vorstellungsgründen und Vitalparametern

In 10 Kliniken ist die standardisierte Dokumentation von Vorstellungsgründen umgesetzt und führt zu plausiblen Ergebnissen. Damit ist die Grundlage für symptombasierte Auswertungen im Gegensatz zu den dominierenden diagnosebasierten Auswertungen gegeben [16]. Die Kombination aus der Erhebung von Vitalzeichen in Abhängigkeit vom Vorstellungsgrund zeigt ein pragmatisches und der Symptomatik entsprechendes Dokumentationsverhalten in den Notaufnahmen. Bestehende Qualitätssicherungsprogramme, wie z. B. für ambulant erworbene Pneumonie [17], würden für entsprechende Vorstellungsgründe eine höhere Dokumentationsrate erwarten lassen, allerdings ist der Vorstellungsgrund „Luftnot“ nicht pathognomonisch für eine Pneumonie. Zusätzlich sind in manchen EDIS konkurrierende Dokumentationsorte für Vitalwerte möglich (z. B. im Ersteinschätzungsmodul und der Verlaufskurve), die von den Schnittstellen teilweise nicht erfasst werden. Hier sind Anpassungen in den User-Interfaces, Erweiterungen der Exportschnittstelle oder auch die Einbeziehung von Subsystemen notwendig.

### Überwachung der Schnittstelle

Die unrealistischen Fallzahlschwankungen in 5 Notaufnahmen sind ein Zeichen dafür, dass der stabile Betrieb von Schnittstellen jenseits verpflichtender bzw. erlösrelevanter Datenerhebung noch nicht flächendeckend gegeben ist. Bei der hier gewählten Lösung der Kopplung eines Registers mit der Routinedokumentation auf Basis von Interoperabilitätsstandards haben sich die Einrichtung der IT-Schnittstelle und deren stabiler Betrieb als zentraler Punkt für den Erfolg des Registers herausgestellt. Die Schnittstellenbetreuung verbraucht damit einen Teil der Ressourceneinsparung durch den Wegfall redundanter Dateneingaben für das Register.

Über das Datenimport-Monitoring ist eine technische Basisüberwachung der Schnittstelle etabliert. Die monatliche Erstellung von klinikinternen Berichten und einrichtungsübergreifenden Benchmark-Berichten erlaubt ein inhaltliches Monitoring relevanter Variablen in den Notaufnahmen. Das Problem der Sicherung und kontinuierlichen Verbesserung der Datenqualität betrifft auch andere, vergleichbare Forschungsdateninfrastrukturen. Die im AKTIN-Projekt genutzte Methode der kontinuierlichen Rückmeldung der Datenqualität an die Anwender wurde auch von anderen Gruppen entwickelt und implementiert [18, 19]. Ein kontinuierliches Feedback der Datenqualität sollte nach den Erfahrungen bei Infrastrukturen zur Routinedatennutzung als State of the Art angesehen werden.

### Limitationen

Das AKTIN-Notaufnahmeregister basiert auf den Inhalten des Datensatzes Notaufnahme; die Dokumentation beschränkt sich derzeit auf die Behandlung in der Notaufnahme [20]. Patientenrelevante Endpunkte, wie Krankenhausverweildauer bei stationärem Aufenthalt und Krankenhausmortalität, lassen sich im Regelbetrieb des Registers noch nicht abbilden. Die Erfassung von Diagnosen folgt in vielen Notaufnahmen den administrativen Vorgaben von Kostenträger und Abrechnungsmodus [15], obwohl die regelhafte Dokumentation einer „unabhängigen medizinischen Notaufnahmediagnose“ nach einheitlicher Systematik wünschenswert wäre.

### Ausblick

Die Architektur mit verteilter Datenhaltung in den Notaufnahmen erlaubt eine zügige Erweiterung des AKTIN-Notaufnahmeregisters um weitere Kliniken. Die Ergänzung um Variablen aus einem folgenden stationären Aufenthalt zur Erfolgskontrolle der Notaufnahmebehandlung steht noch aus. Grundsätzlich ist die Architektur geeignet, auch weitere notfallmedizinische Register mit Daten zu versorgen. Da die gewählten Dokumentationsstandards und IT-Methoden kompatibel zu den Lösungen der Medizininformatik-Initiative (MII) sind, wurde das AKTIN-Notaufnahmeregister als Kooperationspartner der MII aufgenommen. Damit können durch die im Rahmen der MII seit März 2020 verfügbare SNOMED-CT-Lizenz die bisher mangels einer Lizenz verwendeten proprietären Codes umgehend durch diese Terminologie ersetzt werden. Durch die kontinuierliche Datensammlung im lokalen DWH ist es möglich, Datenanforderungen einer tagesaktuellen Gesundheitssurveillance zu bedienen. Nicht zuletzt die aktuelle COVID-19-Pandemie unterstreicht die hohe Bedeutung einer solchen zeitnahen Datenverfügbarkeit. Im März 2020 wurde eine tägliche Datenübermittlung aus den teilnehmenden Notaufnahmen an das Robert Koch-Institut etabliert. Seit dem 24.06.2020 werden dort wöchentliche Notaufnahme-Situationsreports erstellt (www.rki.de/sumo; [2]).

## Fazit für die Praxis

- Die Entwicklung des Datensatzes Notaufnahme der DIVI und Umsetzung in einem internationalen Kommunikationsstandard (HL7 CDA) schafft die Basis für eine vielfältige Datennutzung in Versorgung und Forschung.
- Das AKTIN-Notaufnahmeregister erhebt Versorgungsdaten aller Patienten unabhängig von Kostenträger, Fallart und Abrechnungsmodus.
- Die Zielvorgaben des G‑BA zur Ersteinschätzung bedürfen einer differenzierten Betrachtung und eines Kontextwissens zu den Prozessen und Strukturen der einzelnen Notaufnahmen.
- Die regelhafte Dokumentation von Vorstellungsgründen ist möglich und ermöglicht symptombezogene Analysen.
- Die Daten im AKTIN-Notaufnahmeregister stehen so zeitnah zur Verfügung, dass sie für eine Gesundheitssurveillance nutzbar sind.

## Supplementary Information




