# Supplementary material for: AKTIN – The German Emergency Department Data Registry – real-time data from emergency medicine: Implementation and first results from 15 emergency departments with focus on Federal Joint Committee’s guidelines on acuity assessment
Source: Med Klin Intensivmed Notfmed. 2020 Dec 21;117(1):24–33. [Article in German] doi: 10.1007/s00063-020-00764-2 (PMC7750913; doi:10.1007/s00063-020-00764-2)
Supplement: Supplementary file 1 [file 63_2020_764_MOESM1_ESM.pdf]

Zusatzmaterial

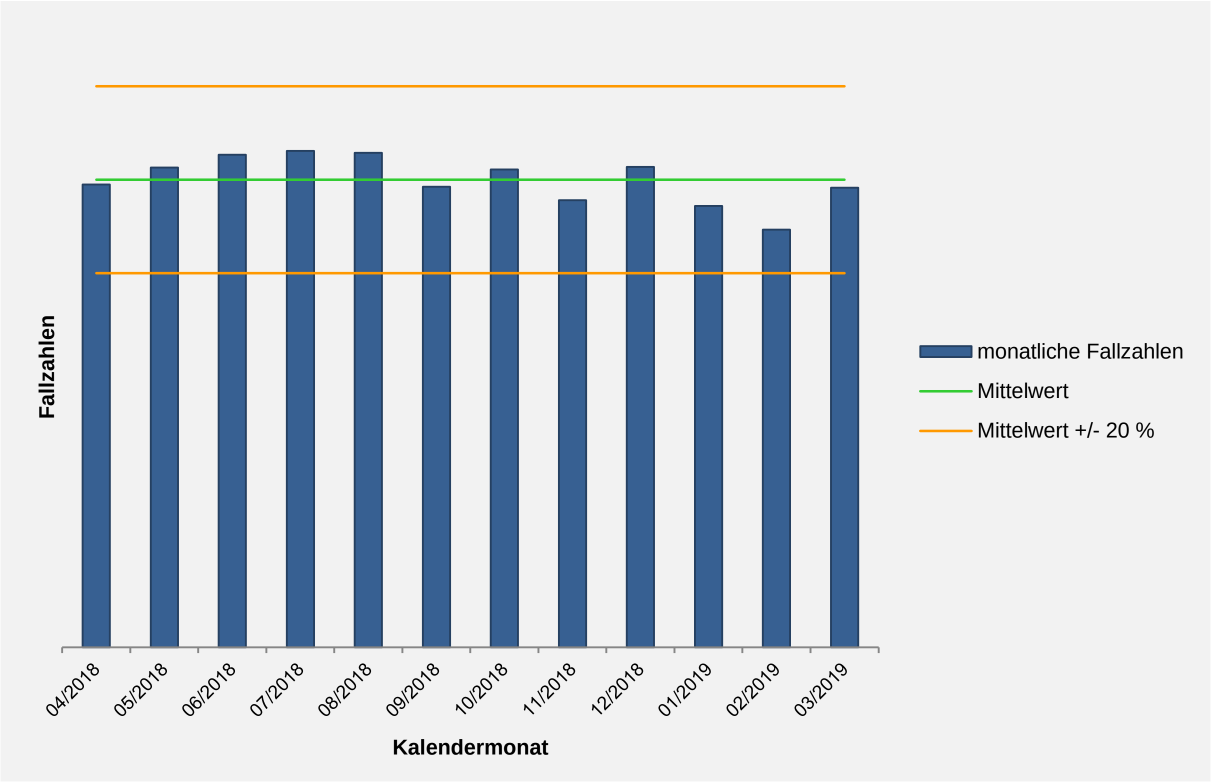

Abb. S1: Exemplarische Notaufnahme mit stabilen Fallzahlen im Zeitverlauf

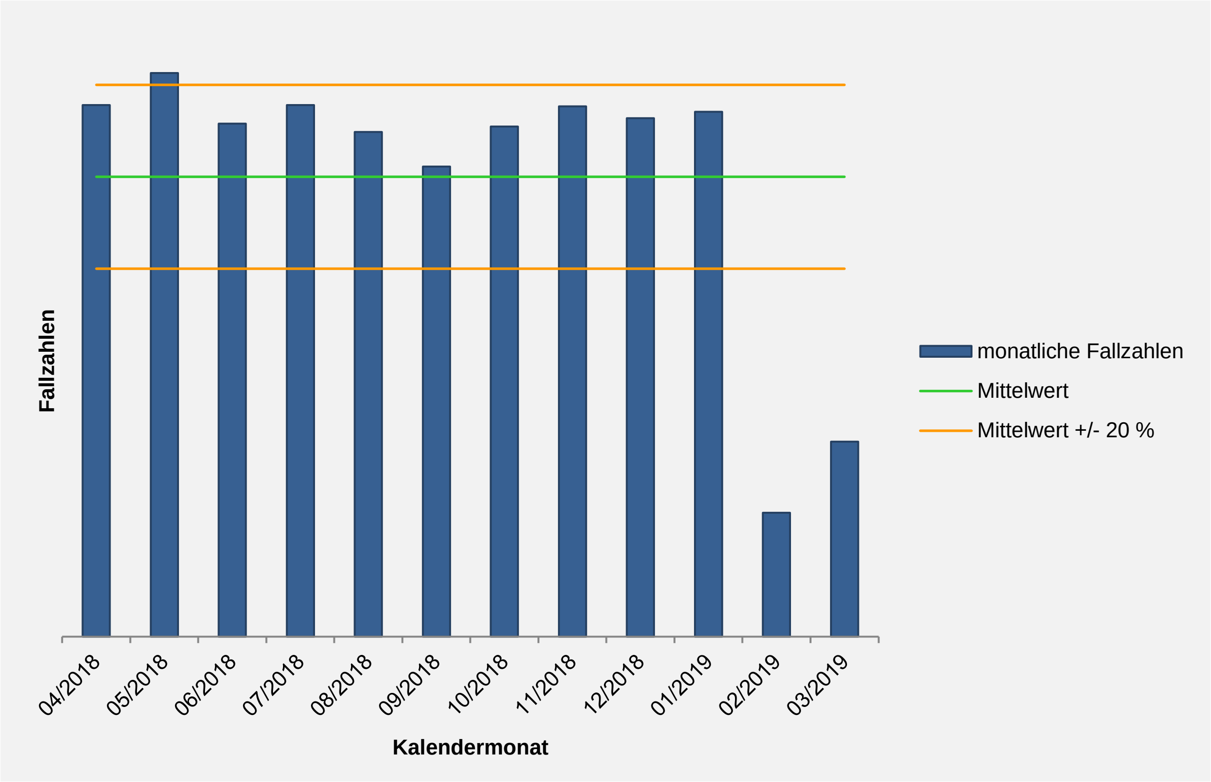

Abb. S2: Exemplarische Notaufnahme mit nicht plausiblen Fallzahlschwankungen im Zeitverlauf
